# Diversity and leaf dry matter yield of Woody forage species in the pastures of South Ari District, Southern Ethiopia

**DOI:** 10.1038/s41598-025-30029-x

**Published:** 2025-11-27

**Authors:** Kuratu Kuke, Yilkal Tadele, Tekle Olbamo

**Affiliations:** 1 Department of Animal Science, Jinka University, Jinka, Ethiopia; 2 Department of Animal Science, Arba Minch University, Arba Minch, Ethiopia

**Keywords:** Composition, Species, Productivity, Productivity, Leaf yield, Ecology, Ecology, Plant sciences

## Abstract

The study was conducted with the objective of assessing fodder tree and shrub species and their foliage yield potentials in private and communal grazing lands across different agroecological environments of South Ari district. A household survey involving 196 respondents was used to identify fodder tree and shrub species and their preferences by livestock species. To evaluate species composition and leaf dry matter yield, a total of thirty-six belt transects (40 m × 40 m for trees and 20 m × 20 m for shrubs) were established. A total of 18 fodder trees and 9 shrubs were identified in the study area, accounting for 79.0% trees and 21.0% shrubs. Among these, 42.1% were categorized as highly desirable, 36.8% as desirable, and 21.1% as less desirable. *Vernonia amygdalina*, *Ficus ovata*, *Dracaena steudneri*, and *Terminalia laxiflora* were the most frequently occurring and widely utilized fodder tree species. Livestock utilized twigs, leaves, fruits, and pods of these species as edible parts. The mean (± S.E.) leaf biomass yields of fodder trees and shrubs were 13.82 ± 0.613 kg/tree and 2.97 ± 0.464 kg/shrub, respectively. Future work should focus on nutritional evaluation and controlled livestock feeding trials to determine the actual feed value and contribution of these species to livestock productivity.

## Introduction

Ethiopia is believed to have the largest livestock population in Africa^1^. The livestock sector contributes substantially to the national economy, accounting for up to 40% of agricultural Gross Domestic Product (GDP), nearly 20% of total GDP, and 20% of foreign exchange earnings in 2017^2^. Despite this importance, livestock production is constrained by inadequate and poor-quality feed resources. According to the Ethiopian Statistics Service^3^, natural pasture and crop residues remain the major feed sources. However, these feed types are typically low in crude protein (CP), digestibility, and mineral concentration, and their availability is often limited^4^.

Feed availability also fluctuates seasonally. During the wet season, natural pasture is the primary feed source across all agroecological environments of Ethiopia^5^. Beyond providing feed, grazing lands also deliver broader benefits, including livelihoods for local communities, habitat for wildlife, ecosystem services, and opportunities for recreation and ecotourism^6^. In the highland areas, natural pastures are dominated by diverse native grasses and legumes, which constitute an important feed base for livestock^7^. Herbaceous pastures lose both biomass and nutritive quality during the dry season and are characterized by low nutrient contents and poor digestibility making them insufficient to meet livestock nutritional requirements^8^. In contrast, woody species (trees and shrubs) remain green during the dry season due to their deep rooting systems and drought tolerance. Their leaves contain higher crude protein and mineral concentrations than herbaceous forages, making them vital for livestock nutrition when grasses decline^9^.

Fodder trees are an important feed source for livestock in a wide range of farming systems across Africa. They are increasingly recognized as a valuable component of animal diets, particularly as a source of protein, in different parts of the world^10^. The edible portions of indigenous fodder trees are leaves, young shoots, twigs, stems, and fruit pods^11^. Beyond their nutritional role, fodder trees offer practical advantages: they are relatively easy to establish, require minimal land, labor, or capital, and can provide fodder within a year of planting^12^. Moreover, trees and shrubs are especially important in arid and semi-arid regions where rainfall is limited and herbaceous feed resources are scarce^13^. The vast majority of Ethiopia’s livestock and grazing land is found in the lowlands^14^. Natural grazing and browsing are primarily based on communal or private lands: communal grazing areas are owned collectively by communities, whereas private grazing or browsing lands belong to individuals, families, or private entities. At present, livestock feeding in the country relies almost entirely on natural grasslands and crop residues^5^.

Several studies on fodder trees have been conducted in different parts of Ethiopia^15^, but these have mainly focused on introduced species such as *Leucaena leucocephala*, *Sesbania sesban*, and *Chamaecytisus palmensis*. In practice, smallholder farmers also depend on indigenous fodder trees and shrubs to supplement livestock diets, particularly during feed shortages. However, information on the availability and yield potential of native fodder tree species remains limited in many parts of the country, and is especially scarce in South Ari District. Therefore, this study was undertaken to assess the availability and foliage yield potential of fodder tree and shrub species across different agroecological environments of South Ari District.

## Materials and methods

### Study area

**Fig. 1:**
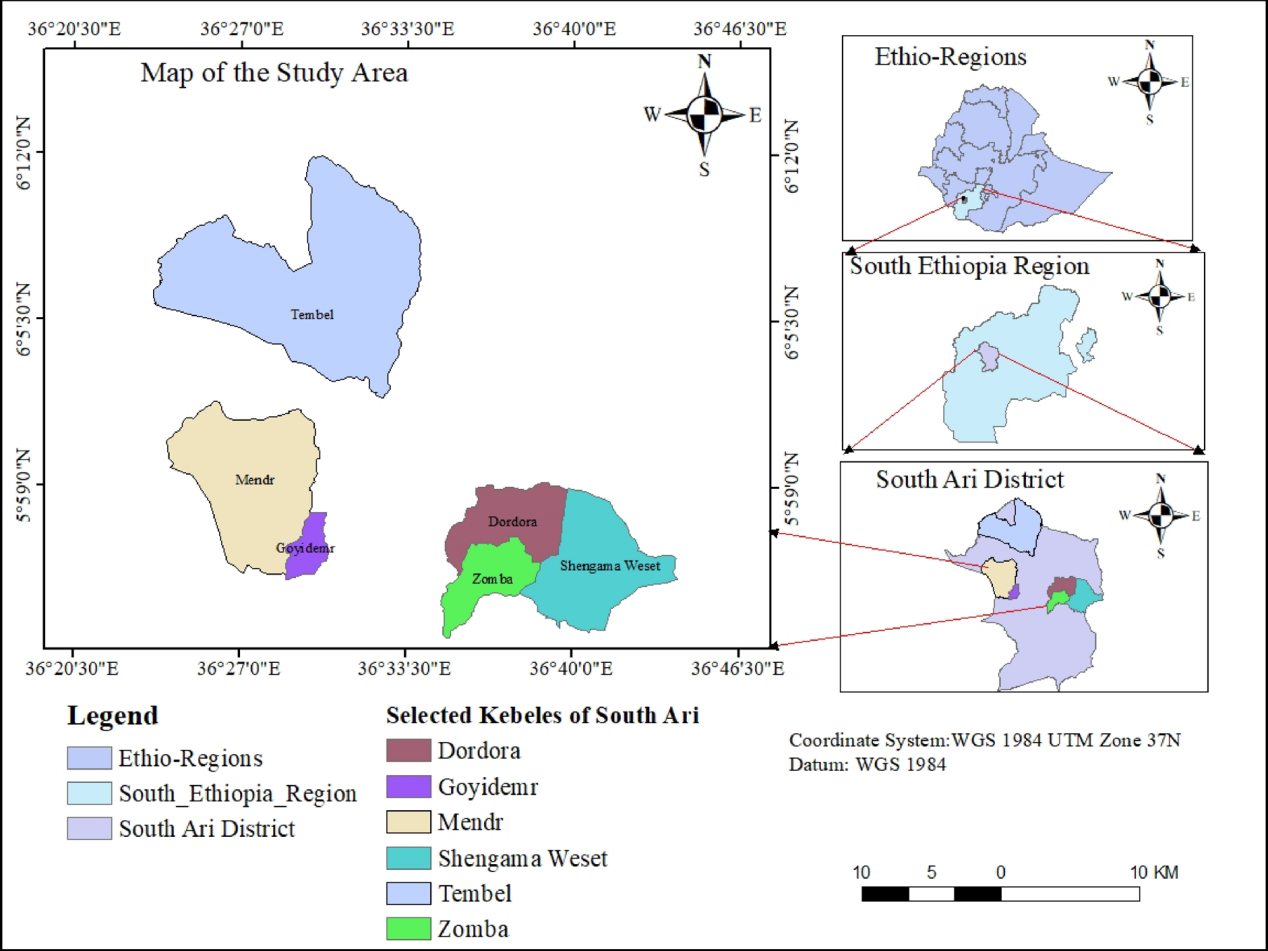
Study area map, generated by the authors using ArcGIS Desktop version 10.8 (Esri, https://www.esri.com.

The study was conducted in South Ari District of Ari Zone (6^0^4’30’’ N; 36^0^30’-37^0^E), Southern Ethiopian Region (Fig. 1). The site altitude ranges from 500 to 3,418 m above sea level. The three agroecological environments of the district are *highland* (20%), *midland* (70%) and *lowland* (10%). The district has a bimodal rainfall pattern with small rainfall from March to May followed by the main rainy season from July to September, with average annual rainfall ranging from 601 to 1600 mm. The average annual temperature ranges between 10.1 °C and 27.5°C^16^.

There are 33 kebeles (28 rural and 5 urban) in the district with altitudes ranging from 500 to 3,418 m above sea level. The farming system is characterized by mixed crop-livestock farming. Regarding the land use, the proportions of cultivated land, grazing land, forest land, cultivable land, non-cultivable land and others are 17, 15.08, 22.43, 8.3, 15.36 and 21.81% respectively^17^. The soils of the south Ari district are spatially variable across the altitudinal gradient, with a predominance of Nitisols in the mid- to high-altitudes^18^.

### Sampling and study site selection

Purposive and random sampling procedures were employed in this study. South Ari District was purposively selected due to its large livestock population and the availability of grazing land. The district was stratified into three agroecological zones based on altitude, following the classification of MoA^19^: highland (2,300–3,418 m a.s.l.), midland (1500–2300 m a.s.l.), and lowland (500–1,500 m a.s.l.).

A total of six kebeles were then randomly selected, proportional to the area coverage of each agroecological environment: Weset and Dordora from the highlands, Zomba and Goydamer from the midlands, and Tembel and Mendri from the lowlands. Within each selected kebele, smallholder farmer households were identified and listed, and respondents were randomly chosen from these lists. The total number of respondents was determined using the probability proportional to size (PPS) sampling technique, and the household sample size for interviews was calculated using the formula:

$$n_{o} = \frac{{Z^{2} *\left( p \right)\left( q \right)}}{{d^{2} }} = 196$$

Where, n_o_= desired sample size determined by the formula of Cochran^20^, when population greater than 10,000; Z = standard normal deviation (1.96 for 95% confidence level); *p* = 0.15 (proportion of population to be included in sample i.e. 15%); q= (1-p) i.e. (0.85); d = is degree of accuracy desired (0.05), with 5% error term. Accordingly, the present study used 196 (173 males and 23 females) randomly selected household respondents from the selected kebeles. Thus, 80, 61 and 55 respondents proportional to its population, respectively from highland, midland and lowland agro-ecological environments were selected and interviewed.

### Data collection methods

Data was collected using questionnaires and field measurements. Land use systems (communal or private grazing) in all agroecological environments (highland, midland and lowland) of the district were considered.

### Questionnaire survey

Structured questionnaire was prepared to identify fodder trees and shrubs utilized by livestock as feed in the study area. Survey respondents categorized tree and shrub forage species into three groups: less desirable, desirable, and highly desirable, based on their importance as livestock feed. In addition, they provided the common names of the plants, identified the plant parts consumed, and specified the types of livestock that utilized them.

### Composition of fodder trees and shrubs

Land use systems (communal and private grazing) were considered across all the three agro-ecological environments of the district (highland, midland, and lowland). Within each combination of land use type and agro-ecological zone, three transects were randomly established. This yielded a total of 36 transects: 18 for trees (40 m × 40 m plots) and 18 for shrubs (20 m × 20 m subplots nested within the tree plots) (Table 1). This approach allowed us to record and enumerates fodder trees and shrubs within each land use system and agro-ecological zone. Because the vegetation in the study area is mixed, comprising both tree-covered and shrubby elements, we used different plot sizes according to plant growth form rather than separating them into distinct vegetation types, following established practices^21^^22^.

**Table 1:** Sampling design showing the distribution of transects by agro-ecological zone and land use system.

Land use type	Highland	Midland	Lowland	Total
Communal grazing	3 transects	3 transects	3 transects	3 transects
Private grazing	3 transects	3 transects	3 transects	3 transects
Subtotal (per trees or shrubs)	6	6	6	18
Total (trees and shrubs)	12	12	12	36

### Leaf yield Estimation of fodder trees and shrubs

In each of the two grazing land uses from the three agro-ecologies, the circumference of the selected trees and shrubs were measured to estimate leaf biomass yield. Measurements were not taken on all woody species encountered but were restricted to major fodder species identified during the survey. These species were chosen because of their palatability, frequency of use as livestock feed, and relative abundance in the study area. The diameters of the plants were calculated using the formula; Diameter at Breast Height = Circumference at Breast Height/ π; where π (pi) is approximately 3.1416. Then, potential leaf biomass yield for each browsing plants were calculated using Petmark^23^ formula: LogW = 2.24 logDT-1.5 (for trees) and LogW = 2.62 logDS-2.46 (for shrubs). Where W = leaf DM yield in kg, DT = the diameter of the trunk (cm) at 1.2 m height (for tree leaf biomass) and DS = the diameter of the stem (cm) at 30 cm height (for shrub leaf biomass).

In addition to woody species (trees and shrubs), the herbaceous species present within each transect were identified and recorded. However, this study focused on forage woody species, as they remain a reliable feed resource for livestock during critical periods of the year.

### Consent to participate and ethical approval

Before the commencement of the research, informed consent to participate was obtained from respondent local farmers and landholders before collecting fodder tree and shrub samples. This study did not involve human participants or experimental animals. All methods were carried out in accordance with Ethiopian National Research Ethics Guidelines^24^. All experimental protocols were reviewed and approved by the Research and Ethics Committee of Jinka University and permission for sample collection was obtained from the South Ari District Agricultural Office.

### Statistical analysis

Statistical Package for Social Sciences (SPSS, version 21) was used for the analysis of collected data after checking and coding. Data were analyzed using a general liner model procedures and Turkey’s mean comparisons were employed. Model: Y_ij_ = µ + α_i_ + Lj + α_i_ *Lj + Σ_ij,,_ Where: Y*ij* = total observation due to i, and j, µ = is overall mean, α_i_ = location (agro-ecology), Lj = land use system (grazing types), Σ_ij_ = random error.

Frequency of species in each transect was calculated using the formula: Frequency (%) = (ni / N) × 100. Where: ni = number of transects in which species i occurs, N = total number of transects sampled. Species were classified by their occurrence frequency as follows: Abundant / Very frequent (> 50%), Frequent / Common (25–50%), Occasional / Less abundant (5–25%), and Rare (< 5%)^25^.

## Results and discussion

### Land holding and use patterns

In the study area, respondents utilized both private and communal grazing lands. The average household landholding is summarized in Table 2. In Ethiopia, land allocation for forage cultivation is generally given the least priority, as increasing population pressure demands land for food crop production and settlement expansion^26^. The present findings reflect this trend, with the lowest land allocation recorded for private grazing land (0.25 ± 0.014 ha), forage cultivation (0.14 ± 0.013 ha), and fodder trees/shrubs (0.07 ± 0.009 ha). These small holdings clearly indicate that land scarcity is a major constraint to livestock productivity in the district.

**Table 2:** Average (Mean ± S.E) land holding/hectare/ of the households in the study area.

Land use type	Agro-ecologies				
	Highland	Midland	Lowland	Overall	*P*-value
Crop land (ha)	1.26 ± 0.019	1.23 ± 0.026	1.27 ± 0.026	1.26 ± 0.013	0.837
Homestead(ha)	0.21 ± 0.033	0.21 ± 0.036	0.21 ± 0.041	0.21 ± 0.021	0.984
Private grazing land(ha)	0.24 ± 0.187	0.23 ± 0.021	0.28 ± 0.034	0.25 ± 0.014	0.080
Forage cultivation land(ha)	0.2 ± 0.018	0.13 ± 0.199	0.15 ± 0.033	0.14 ± 0.013	0.231
Closed plantation land(ha)	0.03 ± 0.004	0.03 ± 0.005	0.03 ± 0.006	0.03 ± 0.003	0.365
Total landholding (ha)	1.96 ± 0.065	1.92 ± 0.064	2.00 ± 0.098	1.96 ± 0.043	0.361

ha = hectare(s), S.E = standard error of mean.

By contrast, Chufa^27^ reported that in Derashe Special District there was no communal grazing land in the mid-altitude areas near the district town, although a few private grazing lands were maintained along the edges of croplands. Similarly, Andualem^28^ indicated that in Essera district of Dawuro Zone, the major sources of natural pasture were communal and private grazing lands, as well as roadside and swampy areas.

### Species diversity in the study grazing lands

In highland of the study area, both herbaceous and woody plant species revealed the highest proportions (*P* < 0.05) than in other agroecological environments (Table 3). A total of 44 herbaceous plant species were identified in the study area, comprising 45.5% Poaceae, 25.0% Fabaceae, and 29.5% Asteraceae. In addition, 18 tree species and 9 shrub species were recorded. Similarly, Alemayehu and Woldegebriel^29^ reported differences in the distribution of herbaceous species across agroecological environments, emphasizing that altitude strongly influences the distribution, growth, and diversity of rangeland plants. Variability in species composition is often shaped by factors such as soil quality, climatic conditions (rainfall and temperature), and management practices, including the grazing system^30^.

**Table 3:** Average herbaceous and Woody species counts of grazing lands.

Proportion (%)	Grazing types	Agro-ecological environments					
		Highland	Midland	Lowland	Overall	P-value	
Herbaceous plants	CG	72.27^a^	70.45^b^	68.18^c^	72.73		0.006
	PG	81.82^a^	79.55^b^	72.27^c^	77.88		0.000
Woody plants	CG	86.84^a^	78.95^b^	77.05^b^	80.78		0.020
	PG	92.11^a^	78.95^c^	81.58^b^	84.21		0.001

Where, superscripts (a, b, c) along row are significantly differed at *P* < 0.05; %=Percentage; CG: communal grazing; PG: private grazing.

### Feeding value and edible parts of selected Woody plants

Fodder trees and shrubs in the study area are particularly important during the dry season, as they provide ruminant livestock with protein- and mineral-rich feed when herbaceous vegetation is scarce. The available trees and shrubs and their edible parts for different livestock species are presented in Table 4. Of the total woody species identified, 42.1% were classified as highly desirable, 36.8% as desirable, and 21.1% as less desirable for livestock forage. In comparison, a study in Assosa Zone reported that of 17 woody species, 53% were highly desirable, 23% desirable, and 22% less desirable^31^. *Oxytenantheria abyssinica* and *Acacia seyal* were the most palatable species, comprising the largest proportion of highly desirable fodder. The selection of indigenous tree and shrub species for fodder production in the dry season is primarily based on species availability and palatability to livestock. Beyond their role as livestock feed, farmers also utilize trees and shrubs for multiple purposes, including income generation through timber sales, fruit production as a source of food, and fuelwood collection^41^. These multiple benefits highlight the integral role of fodder trees and shrubs in smallholder farming systems.

**Table 4:** Edible parts, feed value and livestock species for consuming fodder trees.

Botanical name (scientific)	Vernacular name	Edible parts	Feed value	Livestock species
*Vernonia amygdalina*	*Gara (Girawa)*	Leaf	HD	Cattle, sheep, goat
*Ficus ovate*	*Hom’a(Sholla)*	Leaf, fruit	D	Cattle, sheep, goats
*Dracaena steudneri*	*Wisha*	Leaf	HD	Sheep, goat
*Terminalia laxiflora*	*Sengella*	Leaf, twings	HD	Cattle, sheep
*Ficus sur*	*Shaffa*	Leaf, fruit	D	Cattle, sheep, goat
*Paulownia tomentosa*	*Bata (Bisana)*	Leaf	LD	Cattle, goats
*Moringa stenopetala*	*Shifaraw*	Leaf, twings	D	Cattle, sheep, goat
*Oleaeuropea*	*Weirra (Olive)*	Leaf	LD	Sheep, goat
*Ficus benghalensis*	*Warkka*	Leaf, fruits	D	Cattle, sheep, goat
*Acacia brevispica*	*Ruzza*	Leaf, twings	D	Sheep, goat
*Persea Americana*	*Avocado*	Leaf	HD	Cattle, sheep, goat
*Cordia Africana*	*Ashaa(Wanzza)*	Leaf	D	Cattle, sheep, goat
*Arundinaria alphina*	*Oyssi (Kerkeha)*	Leaf	HD	Cattle, sheep, goat
*Hagenia abyssinica*	*Kosso*	Leaf	HD	Cattle, sheep, goat
*Millettia ferruginea*	*Birrbirra*	Leaf, twings	D	Sheep, goat
*Vachellia farnesiana*	*Chache*	Leaf, twings	LD	Goat, cattle
*Lantana hirta*	*Seki(Gulo)*	Leaf, twings	HD	Cattle, sheep, goat
*Acanthus montanus*	*Komma*	Leaf, twings	HD	Goats

D = desirable, LD = less desirable, HD = highly desirable

The results of this study are consistent with previous reports highlighting the feeding value of indigenous trees and shrubs in various parts of Ethiopia^32^. Livestock utilize multiple parts of browse species, including leaves, pods, twigs, and flowers^33^. Leaves, twigs, and fruit pods of fodder trees are rich in crude protein, minerals, and energy, making them particularly valuable as feed during the dry season^13^. Among these plant parts, leaves are generally the most preferred by livestock. In Alicho-wiriro District, major dry-season feed sources include trees such as *Bundelija polystachya*, *Yushanya alpin*, *Hygenia abysinica*, and *Domoboya torrid*^34^.

### Woody species frequencies of grazing lands in South Ari district

The frequency of occurrence of multipurpose indigenous browse species across different agro-ecologies and land-use units, along with their vernacular and scientific names, is presented in Tables 5 and 6. Among the species identified by interviewed households in the study district, *Vernonia amygdalina*, *Ficus ovata*, *Dracaena steudneri*, and *Terminalia laxiflora* were the most frequently occurring and widely utilized fodder trees. The variation in species frequency across agroecologies may be attributed to differences in altitude, rainfall, and land-use practices.

Similar studies have highlighted the importance of fodder trees and shrubs as livestock feed across different agroecological environments of Ethiopia^35^ For instance, Aynalem^15^ documented 49 fodder tree and shrub species belonging to 43 genera and 31 families across three agroecologies in Arba Minch Zuria Woreda. Their study showed that *Ficus sur* (51.9%), *Ficus sycomorus* (46.5%), and *Mangifera indica* (60.2%) were the most important species in high, middle, and low altitudes, respectively, reflecting the diversity of browse resources available to farmers. In Wolaita Zone, Takele^36^ reported species such as *Acanthus pubescens*, *Buddleja polystachya*, *Celtis africana*, *Combretum molle*, *Millettia ferruginea*, and *Terminalia schimperiana* as key fodder sources.

In the highlands of Sidama region, Adugna^37^ identified *Arundinaria alpine*, *Hygenia abyssinica*, *Erythrina brucei*, and *Vernonia amygdalina* as the most widely utilized fodder trees, while in the midlands, *Cordial africana*, *Millettia ferruginea*, *Erythrina brucei*, and *Dracaena steudneri* were dominant. In the Mid Rift Valley, Shenkute^13^ reported *Acacia tortilis*, *Ficus gnaphalocarpa*, *Balanites aegyptica*, *Dichrostachys cinerea*, and *Grewia bicolor* as the most frequently used browse species.

**Table 5:** Frequency of fodders trees in grazing lands of the study area.

Botanical name (scientific)	Vernacular name *(Arigna)*	Family name	Land use pattern	Agro-ecologies			Over all	P-values
				Highland	midland	lowland		
*Vernonia amygdalina*	*Gara (Girawa)*	*Asteraceae*	CG	20.67	21.00	17.88	19.85	0.312
			PG	17.57	19.96	18.95	18.83	0.055
*Ficus ovate*	*Hom’a(Sholla)*	*Moraceae*	CG	16.33^b^	18.10^a^	15.20^c^	16.54	0.010
			PG	16.11	14.33	16.21	15.55	0.222
*Dracaena steudneri*	*Wisha*	*Asparagaceae*	CG	13.46^b^	12.33^c^	13.82^a^	13.20	0.002
			PG	14.50^a^	12.98^c^	13.56^b^	13.68	0.001
*Terminalia laxiflora*	*Sengella*	*Combretaceae*	CG	9.56^b^	7.69^c^	10.27^a^	9.17	0.000
			PG	11.00^b^	10.33^c^	12.26^a^	11.19	0.000
*Ficus sur*	*Shaffa*	*Moraceae*	CG	8.56^b^	5.91^c^	8.80^a^	7.76	0.002
			PG	9.50	8.98	9.11	9.20	0.055
*Paulownia tomentosa*	*Bata (Bisana)*	*Paulowniaceae*	CG	6.22^b^	5.56^c^	7.60^a^	6.46	0.000
			PG	7.13^b^	7.27^a^	5.55^c^	6.65	0.000
*Moringa stenopetala*	*Shifara*	*Moringaceae*	CG	5.05^b^	5.33^b^	6.93^a^	5.77	0.000
			PG	6.00^b^	7.00^a^	5.22^c^	6.07	0.000
*Oleaeuropea*	*Weirra (Olive)*	*Oleaceae*	CG	4.00^c^	4.67^b^	4.78^a^	4.48	0.000
			PG	3.90^c^	5.77^a^	4.94^b^	4.87	0.005
*Ficus benghalensis*	*Warkka*	*Moraceae*	CG	3.73^c^	4.56^a^	3.78^b^	4.02	0.000
			PG	3.63^c^	4.33^a^	4.07^b^	4.01	0.000
*Acacia brevispica*	*Ruzza*	*Fabaceae*	CG	3.00^b^	4.27^a^	2.96^c^	3.41	0.000
			PG	2.82	2.27	3.30	2.79	0.052
*Persea Americana*	*Avocado*	*Lauraceae*	CG	2.80	3.22	2.45^c^	2.82	0.200
			PG	2.56	2.23	2.19	2.33	0.883
*Cordia Africana*	*Ashaa(Wanzza)*	*Boraginaceae*	CG	1.57^b^	1.56^c^	2.37^a^	1.83	0.000
			PG	1.98^a^	1.34^c^	1.81^b^	1.71	0.000
*Arundinaria alphina*	*Oyssi (Kerkeha)*	*Poaceae*	CG	2.05	2.78	1.83	2.22	0.060
			PG	1.76^a^	1.21^c^	1.55^b^	1.51	0.002

Means with different superscripts (a, b, c) along row are significantly differed at *P* < 0.05; %: Percentage; CG: communal grazing, PG: private grazing, Frequency: >50% = Abundant, 25–50% = Frequent, 5–25% = Less abundant, and < 5% = Rare.

**Table 6:** Frequency (%) of fodder shrubs in grazing lands of the study area.

Botanical name	Vernacular name	Land use	Agro-ecologies				*P*-value
			Highland	Midland	Lowland	Over all	
*Millettia ferruginea*	*Birrbirra*	CG	26.98^a^	27.67^b^	26.00^c^	26.88	0.001
		PG	25.50	27.83	26.44	26.59	0.300
*Vachellia farnesiana*	*Chache*	CG	23.67	25.88	25.25	24.93	0.071
		PG	24.33^c^	24.56^b^	26.33^a^	25.07	0.000
*Lantana hirta*	*Seki(Gulo)*	CG	21.58^c^	22.00^b^	24.40^a^	22.66	0.000
		PG	22.60^a^	20.00^c^	21.68^b^	21.43	0.000
*Acanthus montanus*	*Komma*	CG	18.37	16.33	20.22	18.31	0.052
		PG	17.33^c^	19.94^a^	17.50^b^	18.26	0.000
Others		CG PG	9.40 10.24	8.12 7.67	4.13 8.05	7.22 8.65	NS NS

Means with different superscripts (a, b, c) along row are significantly differed at *P* < 0.05; NS: Non-significant at *P* > 0.05; %: Percentage; CG: communal grazing, PG: private grazing, Frequency: >50% = Abundant, 25–50% = Frequent, 5–25% = Less abundant, and < 5% = Rare.

### Biomass yield from fodder trees in the study area

The results showed that the average (mean ± SE) biomass yield of fodder trees from communal and private grazing lands was 13.19 ± 0.534 kg DM/tree and 14.45 ± 0.692 kg DM/tree, respectively (Table 7). Overall, the biomass yield of fodder trees ranged from 2.10 ± 0.088 to 33.42 ± 0.630 kg DM/tree across the three altitudinal zones. Fodder trees and shrubs in the study district thus represent a significant feed resource for livestock production. Notably, dry matter production of fodder trees and shrubs was significantly higher (*P* < 0.05) in the lowland areas compared to midland and highland zones.

In contrast, Takele^36^ reported higher biomass yields of 24.55–958.76 kg DM/tree for selected indigenous fodder trees and shrubs in Wolaita Zone, southern Ethiopia. They also observed total biomass dry matter production of 74.36–100 kg DM/ha and 500–800 kg DM/ha on cultivated and uncultivated land, respectively. Similarly, Adugna^37^ reported average DM biomass yields of 0.22–15.43 kg/tree, 0.28–23.1 kg/tree, and 10.81–54.1 kg/tree for highland, midland, and lowland areas, respectively.

**Table 7:** Foliage biomass (Mean ± SE) yield (kgDM) of selected fodder trees (120 cm) height.

Fodder species	Land use	Agro-ecologies				*P*-value
		Highland	Midland	Lowland	Overall	
*Vernonia amigdalina*	CG	15.24 ± 0.032^c^	19.95 ± 0.122^b^	26.82 ± 0.346^a^	17.01 ± 0.167	0.000
	PG	18.60 ± 0.230^c^	23.00 ± 0.086^b^	28.01 ± 0.174^a^	18.54 ± 0.163	0.002
*Ficus ovate*	CG PG	21.22 ± 0.744^c^ 19.78 ± 0.422	24.20 ± 0.406^b^ 28.23 ± 0.242	30.36 ± 0.083^a^ 29.20 ± 0.259	22.26 ± 0.411 25.74 ± 0.308	0.020 0.301
*Terminalia axiflora*	CG	9.02 ± 0.144^c^	10.50 ± 0.504^b^	12.87 ± 0.129^a^	10.80 ± 0.259	0.005
	PG	12.30 ± 0.531	16.15 ± 0.033	17.50 ± 0.354	12.01 ± 0.306	0.065
*Dracaena steudneri*	CG	10.31 ± 0.056	13.90 ± 0.544	18.93 ± 0.371	10.05 ± 0.324	0.254 0.622
	PG	11.05 ± 0.125	15.30 ± 0.110	16.12 ± 0.133	10.06 ± 0.123	
*Ficus sur*	CG PG	13.11 ± 0.150 12.87 ± 0.267	14.00 ± 0.089 12.33 ± 0.234	19.45 ± 0.047 13.00 ± 0.055	13.52 ± 0.095 12.73 ± 0.185	0.100 0.078
*Paulownia tomentosa*	CG PG	2.10 ± 0.088 6.73 ± 0.433	7.00 ± 0.910 5.14 ± 0.066	8.62 ± 0.455 8.04 ± 0.303	5.24 ± 0.484 6.97 ± 0.267	0.204 0.082
*Moringa stenopetala*	CG PG	6.90 ± 0.370^c^ 8.07 ± 0.771	8.30 ± 0.323^b^ 9.06 ± 0.069	11.42 ± 0.114^a^ 14.07 ± 0.037	7.37 ± 0.269 8.40 ± 0.192	0.000 0.300
*Oleaeuropea*	CG PG	8.93 ± 0.328 9.01 ± 0.550^b^	6.65 ± 0.045 8.77 ± 0.300^c^	8.65 ± 0.145 9.77 ± 0.163^a^	8.08 ± 0.273 8.52 ± 0.338	0.205 0.000
*Ficus benghalensis*	CG PG	8.33 ± 0.190^b^ 6.60 ± 0.098^c^	7.35 ± 0.212^c^ 7.11 ± 0.087^b^	9.04 ± 0.053^a^ 8.38 ± 0.127^a^	7.56 ± 0.152 7.36 ± 0.104	0.000 0.050
*Solanum incanum*	CG PG	7.11 ± 0.101^b^ 4.55 ± 0.562^c^	9.46 ± 0.038^a^ 5.82 ± 0.090^b^	6.54 ± 0.085^c^ 6.88 ± 0.110^a^	6.04 ± 0.075 5.75 ± 0.254	0.001 0.000
*Acacia brevispica*	CG PG	10.80 ± 0.443^c^ 12.09 ± 0.070	16.98 ± 0.033^b^ 10.10 ± 0.050	18.11 ± 0.056^a^ 13.53 ± 0.074	9.63 ± 0.177 10.91 ± 0.065	0.020 0.724
*Persea Americana*	CG PG	15.50 ± 0.260^b^ 22.42 ± 0.600^c^	13.36 ± 0.344^c^ 33.42 ± 0.630^a^	15.72 ± 0.130^a^ 25.34 ± 0.165^b^	13.86 ± 0.245 19.40 ± 0.465	0.000 0.006
*Arundinaria alpina*	CG PG	9.09 ± 0.220^a^ 14.18 ± 0.177^c^	8.74 ± 0.072^c^ 16.30 ± 0.119^b^	10.98 ± 0.036^b^ 17.56 ± 0.244^a^	8.60 ± 0.109 12.41 ± 0.180	0.001 0.000
*Hagenia abyssinica*	CG PG	8.83 ± 0.089^c^ 8.06 ± 0.762	9.31 ± 0.034^a^ 8.43 ± 0.226	9.02 ± 0.343^b^ 9.06 ± 0.191	8.28 ± 0.155 11.54 ± 0.393	0.000 0.223
*Cordia Africana*	CG PG	23.44 ± 0.078^a^ 22.70 ± 0.023	22.86 ± 0.080^b^ 21.35 ± 0.303	19.10 ± 0.402^c^ 24.23 ± 0.077	20.47 ± 0.187 22.78 ± 0.134	0.043 0.601

Where; Superscripts (a, b, c) along row show significant difference at *P* < 0.05; SE: Standard Error; NS: Non-significant at *P* > 0.05; kg/tree: kilogram of dry matter per tree.

Jabesa^32^ documented biomass yields of 7.98–19.78 kg/tree in midland and 9.87–178.06 kg/tree in highland agroecologies of Kellem Wolega, Ethiopia. In Arba Minch, Habtamnesh and Agena^38^ reported leaf yields of 25.92 kg DM/tree for *D. giganteus*, 19.60 kg DM/tree for *B. aegyptiaca*, and 22.53 kg DM/tree for *T. brownie*. Leaf biomass yields reported for different fodder tree species in Wolaita Zone were 958.76 kg/tree for *Erythrina brucei*, 925.53 kg/tree for *Cordial africana*, 95.577 kg/tree for *Vernonia amygdalina*, 96.539 kg/tree for *Erythrina cymosa*, 39.60 kg/tree, and 9.12 kg/tree for *Dracaena abyssinica*^36^. Similarly, Getachew and Wondimu^39^ recorded dry matter yields of 40.82 kg/tree for *Chamaecytisus palmensis* and 317.18 kg/tree for *Erythrina brucei* in Hadiya and Kembata-Tembaro Zones, Southern Ethiopia. Variations in dry matter yield among species are likely due to differences in agroecology, soil, growth traits, harvest stage, season, and environmental factors.

### Dry matter yield of shrubs in the study area

The biomass yield of selected indigenous fodder shrubs across the three agroecologies is presented in Table 8. The results indicate that the mean (± SE) biomass yield of fodder shrubs ranged from 1.55 ± 0.114 to 4.53 ± 0.386 kg/shrub across the three agroecological zones and between the two land-use types.

**Table 8:** Foliage biomass (Mean ± SE) yield (kg DM) of selected shrubs (30 cm) height.

Fodder species	Land use	Agro-ecologies				*P*-value
		Highland	Midland	Lowland	Overall	
*Millettia ferruginea*	CG	3.15 ± 0.400^a^	2.42 ± 0.088^c^	3.00 ± 0.557^b^	2.86 ± 0.348	0.000
	PG	3.67 ± 0.332^c^	4.49 ± 0.601^a^	3.72 ± 0.402^b^	3.96 ± 0.445	0.000
*Vachellia farnesiana*	CG PG	1.59 ± 0.118^c^ 2.34 ± 0.690^b^	2.12 ± 0.446^b^ 1.55 ± 0.114^c^	2.33 ± 0.207^a^ 2.56 ± 0.611^a^	2.01 ± 0.257 2.15 ± 0.472	0.000 0.000
*Lantana hirta*	CG PG	2.21 ± 0.301 1.88 ± 0.122^c^	2.38 ± 0.042 2.15 ± 0.400^b^	3.03 ± 0.434 2.24 ± 0.550^a^	2.54 ± 0.259 2.09 ± 0.357	0.073 0.012
*Acanthus montanus*	CG PG	3.13 ± 0.200 4.47 ± 0.466^c^	2.00 ± 0.160 3.66 ± 0.034^b^	3.12 ± 0.322 4.53 ± 0.386^a^	2.75 ± 0.227 4.22 ± 0.295	0.551 0.000

Superscripts (a, b, c) along row show significant difference at *P* < 0.05; SE: Standard Error; kg/ shrub: kilogram of dry matter per shrub.

In Kellem Wollega Zone, Western Ethiopia, Jabesa^32^ reported biomass yields of 1.6, 1.34, 1.82, and 3.6 kg DM/shrub for *Rhoicissus tridentata*, *Ricinus communis*, *Coronopus didymus*, and *Acanthus polystachius*, respectively. Similarly, Ararsa^41^ recorded dry leaf yields of 1.51, 2.36, 2.06, and 1.18 kg DM/shrub for *Rhoicissus tridentata*, *Combretum paniculatum*, *Ricinus communis*, and *Ficus palmata* Forsk, respectively.

## Conclusion

Communal and private grazing lands were the dominant land use systems across the three altitudinal zones of the study area. *Vernonia amygdalina*, *Ficus ovata*, *Dracaena steudneri*, *Terminalia laxiflora*, and *Ficus sur* were the most popular forage resources identified in the district. These species are well adapted to the local environment and contribute significantly to feed availability, with leaf dry biomass yields ranging from 5 to 22 kg per tree for fodder trees and 2–4 kg per shrub.

From the findings, we recommend that the fodder resources identified across different land-use types and agroecological environments should be widely promoted and demonstrated to address the prevailing feed shortages. Farmers’ knowledge and skills in the management of fodder trees and shrubs need to be strengthened through training and extension support. Furthermore, comprehensive evaluations of the nutritional composition and feeding value of these species should be carried out under controlled animal experiments.

## Data Availability

Data are available from the authors upon reasonable request.
